# The Synergic Effect of Tubal Endometriosis and Women’s Aging on Fallopian Tube Function: Insights from a 3D Mechanical Model

**DOI:** 10.3390/bioengineering11080852

**Published:** 2024-08-20

**Authors:** Mayssam Nassir, Mattan Levi, Natan T. Shaked

**Affiliations:** Department of Biomedical Engineering, Faculty of Engineering, Tel Aviv University, Tel Aviv 69978, Israel; mayssam.nassir@gmail.com (M.N.); mattanlevi@gmail.com (M.L.)

**Keywords:** fallopian tube, endometriosis, women’s aging, sperm and oocyte dynamics, infertility, finite element modeling, biomechanical modeling

## Abstract

The fallopian tubes are essential for human fertility, facilitating the movement of sperm and oocytes to the fertilization site and transporting fertilized oocytes to the uterus. Infertility can result from changes in the fallopian tubes due to tubal endometriosis and women’s aging. In this study, we modeled human fallopian tubes with and without endometriosis for different women’s age groups to evaluate the chances of normal sperm cells reaching the fertilization site and oocytes arriving at the uterine cavity. For this purpose, we employed a distinctive combination of simulation tools to develop a dynamic three-dimensional (3D) model of normal human sperm cells and oocytes swimming inside normal and endometriosis-affected human fallopian tubes for different women’s group ages. We observed that in tubal endometriosis cases, fewer sperm cells reach the fertilization site and more oocytes become trapped in the tube walls compared to normal tubes. Additionally, aging decreases the number of sperm cells and oocytes reaching the fertilization site in normal and endometriosis-affected tubes. Our model evaluates the mechanisms of sperm and oocyte behaviors due to women’s aging and fallopian tube issues caused by endometriosis, presenting new avenues for developing diagnostic and treatment tools for tubal endometriosis and age-related infertility issues.

## 1. Introduction

Successful natural pregnancy involves the effective transport of sperm, oocyte, and embryo through the fallopian tubes. The propulsion inside the fallopian tube is achieved by the interactions between the ciliary beat, mucus flow, peristaltic contraction, and the complicated morphology of the fallopian tube [1,2]. Several studies focused on the main functions of the fallopian tube and its influence on conception, successful pregnancy, and fertility [3,4,5,6]. Fallopian tube function is essential for natural fertilization and is a critical factor in female fertility. In the last decade, numerous pathological situations related to infertility due to fallopian tube diseases and disorders have been studied [7]. These studies concluded that a damaged fallopian tube is one of the most common reasons for female fertility problems requiring infertility treatments [8,9,10,11,12].

The fallopian tubes are tubular, seromuscular organs connecting the ovaries and the uterine fundus in the female pelvis [13]. They consist of four portions: intramural, isthmus, ampulla, and infundibulum [1,14,15]. The intramural and isthmus portions are short and straight tubes connecting the uterus with the ampulla. Fertilization occurs in the ampulla, which is the longest portion with high-density ciliated cells. The inner surface of the fallopian tube is called the mucosal layer, and it contains longitudinal cilia which facilitate the movement of sperm cells and oocytes [16,17,18]. Uterine peristalsis results from the contractions of the muscular fibers enabling the bidirectional transport of the sperm cells and oocytes [19,20]. Tubal motility facilitates the progress of the sperm cells toward the ovary (pro-ovary) and the oocyte movement before and after fertilization on the opposite side (pro-uterus). The sperm and the oocyte meet at the fertilization site due to the temporally coordinated process of the tubal contractions [21,22]. Several studies suggested that uterine contraction is an important mechanical factor for fertility; however, the mechanism that controls the coordination and activity of the contraction is still unclear [23,24,25].

Tubal-factor infertility can occur due to disorders in the fallopian tube caused by inflammation, infection, cancer, endometriosis, or women’s aging. Biological, physical, and morphological changes are induced in the fallopian tube due to women’s aging, causing loss of reproductive capacity [26,27,28,29,30,31,32,33,34]. Moreover, the density of the tubal ciliated cells decreases with aging, as well as the elasticity of the mucosal layer [32,33,34,35,36,37]. Therefore, various studies have investigated the influence of these age-related changes in the fallopian tube on female fertility [26,30,31,37]. Fertility problems in women can also be detected in chronic conditions in the fallopian tube, like tubal endometriosis. Tubal endometriosis is a chronic benign gynecological disease that is characterized by endometria deposits mainly within any part of the fallopian tube. Epithelial and stromal cells, like endometrium, exist in ectopic sites outside of the internal lining of the uterine cavity and cause endometriosis [38,39,40,41]. A few studies have focused on reconstructing 3D geometrical models of human organs to provide a better understanding of their functionality and physiology. For example, one study utilized advanced stereographic computer technology to investigate the three-dimensional (3D) architecture of peritoneal endometriosis [42]. Impairment in the fallopian tube’s main functions due to endometriosis causes damage to the sperm-carrying capacity and oocyte transportation, which reduces fertilization and successful pregnancy rates [43,44,45]. A previous study developed a 3D finite element model of the human endometrium and embryo, demonstrating the critical impact of impaired interaction between the implanting blastocyst and endometrium on outcomes such as various pregnancy complications [46]. Another study introduced a computational model designed to explore the influence of uterine fluid pressure on the progression of endometriosis, providing insights into its potential implications for fertility and pregnancy [47].

Endometriosis causes a 40–80% decline in percent progressive motile sperm according to its severity, and it could be a reason for fertility problems in some couples [41,48,49]. Tubal endometriosis can also impede the oocyte inside the fallopian tube and prevent its meeting with a sperm cell [50]. If fertilization occurs, the fertilized egg can get stuck inside the damaged fallopian tube on its way to the uterus body, which is known as ectopic pregnancy [51]. The risk of ectopic pregnancy in women in their 20s is about 2% and rises steeply as the women’s age increases above 25 years [52,53]. Moreover, based on meta-analysis studies, the risk of ectopic pregnancy increases approximately 2.6 times in a fallopian tube with endometriosis [51,54]. Hence, tubal endometriosis can be a significant symptom of sperm motility reduction, fertilization failure, and ectopic pregnancy, especially with women’s aging.

In the present study, we have developed a dynamic 3D mechanical model of sperm cells and oocytes swimming inside healthy fallopian tubes and tubal endometriosis in different age groups. We created a 3D model for oocytes and simulated their transport through fallopian tubes in different women’s age groups based on geometrical models developed in our previous studies [55]. Additionally, we developed three 3D models of fallopian tubes with tubal endometriosis for each women’s age group. These models incorporated ciliary activity and uterine peristalsis to mimic the biomechanics of fallopian tube walls. Through mathematical and mechanical simulations, we analyzed sperm cells and oocyte dynamics in normal and endometriosis-affected tubes across different women’s age groups. The study evaluated the percentage of sperm cells reaching fertilization sites and oocytes reaching the uterine cavity after 80 h, shedding light on the impact of tubal endometriosis and women’s age on sperm and oocyte behaviors within the fallopian tube and providing insights into fertility rates and the mechanisms underlying infertility due to tubal endometriosis.

## 2. Methods and Materials

### 2.1. 3D Modeling of the Human Fallopian Tubes

**Figure 1:**
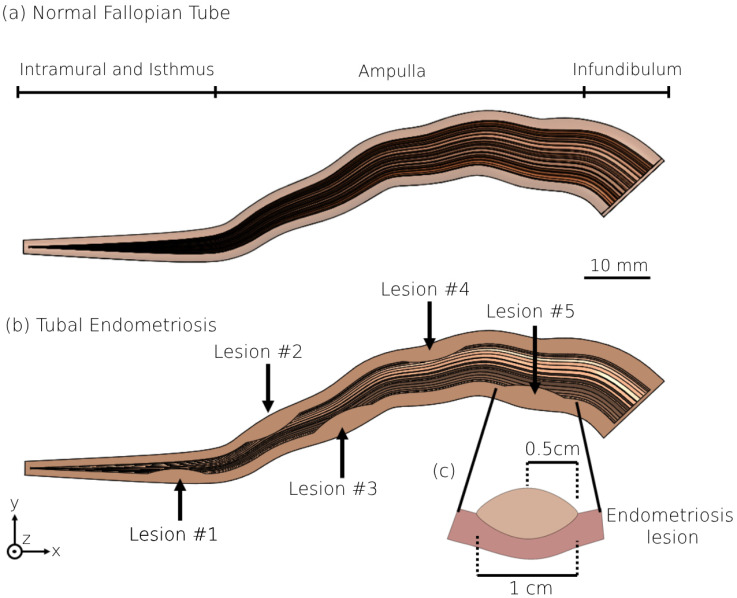
The 3D models of normal fallopian tube and tubal endometriosis. The 3D geometrical model of (**a**) the normal fallopian tube, and (**b**) the fallopian tube with five endometriosis lesions for women in their 20s. (**c**) Detailed geometric model of an individual endometriosis lesion.

We employed the 3D geometrical models of the human fallopian tube developed in our previous study for women in their 20s, 30s, and 40s [56]. The geometrical values of each fallopian tube were based on magnetic resonance imaging (MRI) results of the human fallopian tube [57,58,59,60,61] and studies on the morphological changes in the human uterine tube with aging [26,27,28,29,30,31,62]. Each fallopian tube model contains a straight part, intramural and isthmus, and a convoluted site with a high concentration of ciliated cells, the ampulla. The 3D models exhibit a tubular structure with radial tapering in the intramural region (see Figure 1a). The inner layer of the fallopian tube model is composed of internal longitudinal folds with ciliated cells to describe the mucosal layer. The morphology of the inner layer changes from a columnar to cuboidal shape according to the different age groups. The geometrical properties of the various parts of the fallopian tube models differ based on women’s ages [56]. The models were built in SolidWorks (Premium 2022 x64 Edition, Waltham, MA, USA).

### 2.2. 3D Modeling of the Human Fallopian Tubes with Endometriosis

**Figure 2:**
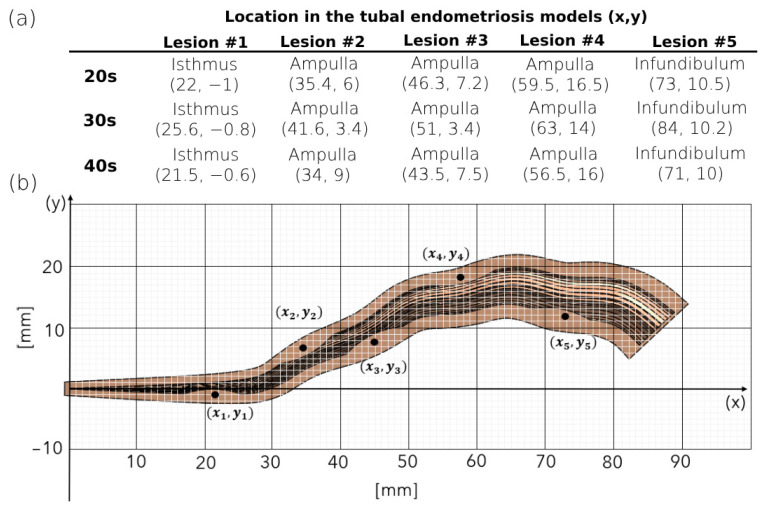
Lesion locations in the tubal endometriosis models. (**a**) The locations of the lesions in the tubal endometriosis models for women in their 20s, 30s, and 40s. (**b**) The coordinates of lesions in the tubal endometriosis.

We also created three 3D geometrical models of fallopian tubes with endometriosis for each women’s age group. These tubal endometriosis models were built by incorporating the geometrical specifications of the normal fallopian tube models, as well as introducing five lesions of uniform size and shape to simulate endometriosis. In each tubal endometriosis model, a single lesion is located at the isthmus region and four lesions are distributed equidistantly within the ampulla, each 17 mm apart (see Figure 1b). The endometriosis lesion is approximated by an elliptical shape, 1 cm long and 0.5 cm wide (see Figure 1c). The coordinates of the lesions in the different tubal endometriosis models are presented in Figure 2. The number of endometriosis lesions was chosen to represent an advanced stage of tubal endometriosis disease. We strategically placed these lesions in various areas on both sides of the fallopian tube to cover all sections, especially the transport tube (the intramural and isthmus portions) and the fertilization site. Testing three other models of 3, 7, and 10 lesions in different places (see Figure S1) led to similar results and conclusions.

### 2.3. Peristalsis–Ciliary Flow

We presented ciliated cells as 3D flexible cylinders distributed across the mucosal layer of the fallopian tube models. The ciliated cell density decreases as the woman ages, 2000 $\left[\frac{1}{{mm}^{2}}\right]$ for women in their 20s, 1600 $\left[\frac{1}{{mm}^{2}}\right]$ for women in their 30s, and 1400 $\left[\frac{1}{{mm}^{2}}\right]$ for women in their 40s [33,63]. An orthogonal force was applied to each cilia model to perform the cilia beat frequency of 5.4 [Hz] as reported in earlier studies [64,65]. We also applied uniformly distributed forces on the mucosal layer of each fallopian tube to simulate the peristalsis waves. The forces are bidirectional, initiated in the intramural portion and propagating towards the ampulla, and from the infundibulum region towards the isthmus portion. The cross-sections of the fallopian tube model were subjected to vertical force as a function of distance and time. Contraction intensity of 20 [mmHg] and frequency of 1.5 $\left[\frac{1}{min}\right]$ were determined based on previous studies about uterine peristalsis [23,66,67]. Video S1 shows a single tubal contraction of the fallopian tube model for women in their 20s. We assumed that the mucosal layer is made of isotropic compressible materials and used a neo-Hookean constitutive model with strain energy function:

(1) $$W=\frac{\mu }{2}\left(I_{1}-3\right)+\mu lnJ+\;\frac{\lambda }{2}{\left(lnJ\right)}^{2},$$

(2) $$\lambda =\;\frac{\nu L*\kappa }{\left(1+\nu \right)\left(1-2\nu \right)*A},$$

(3) $$\mu =\;\frac{L*\kappa }{2A(1+\nu )},$$

where $I_{1}$ is the first invariant of the right Cauchy–Green deformation tensor, J is the determinant of the deformation gradient tensor, μ and λ are the Lamé parameters that relate to stiffness (κ=12–14 kPa), L=85–105 mm is the length, $A=5–40\;{mm}^{2}$ is the area, and ν=0.49  is the Poisson’s ratio of the muscle layer [68,69].

### 2.4. 3D Modeling of the Female Oocyte

**Figure 3:**
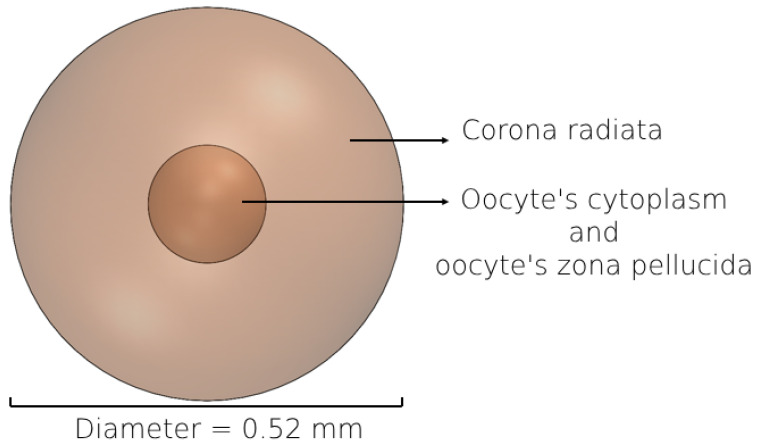
Geometric model of female oocyte: 3D representation featuring cytoplasm, zona pellucida, and corona radiata.

We constructed a 3D geometrical model representing a normal female oocyte to analyze its movement inside the different models of the fallopian tube. The 3D morphology of the oocyte model was determined based on the imaging results of previous studies [70,71,72,73]. The oocyte model approximates a sphere shape, consisting of cytoplasm and zona pellucida with a diameter of 0.12 mm and corona radiata with a diameter of 0.4 mm (Figure 3) [74,75,76,77]. The oocyte is released from the ovary enveloped by corona radiata cells, which impart an outer roughness. Approximately 12 h later, these cells shed, and the morphology of the oocyte tends to become more spherical in shape. Given the 80 h simulation duration, during which the oocyte is predominantly devoid of corona radiata cells, we assumed it to be spherical for most of the time. The mechanical characteristics of the female oocyte model such as elasticity (10 ± 5 kPa) were taken from previous studies about the female oocyte biomechanics [77,78,79].

### 2.5. 3D Human Sperm Model

We used the numerical mechanical model of normal sperm cells developed in our previous study [55,80]. The 3D geometrical structure is based on previous dynamic 3D optical imaging experimental results of sperm cells by our group [81]. The sperm model includes a head, flagellum, and midpiece [55,56,80]. The head of the model is approximated by an elliptical shape, 4.2 µm long and 2.85 µm wide with radial symmetrical tapering reaching the tip of the head, approximated as 0.46 µm. The midpiece contains a filament structure surrounded by spiral arrays of mitochondria, 4 µm in length and 1 µm in diameter. The flagellum comprises a 3D flexible filament with a length of 55 µm, where its diameter decreases from 1 µm to 0.1 µm at the distal end [82,83].

### 2.6. Beat Pattern

To perform dynamic simulations, we meshed the 3D geometrical sperm models using the generation and processing toolbox in SolidWorks to create a 3D element mesh. Each element is described as a 3D continuous, parabolic tetrahedral solid element and treated as an independent body based on the finite element method. Along the tail filament, 100 solid tetrahedral elements were positioned at equal intervals to represent dynein motor proteins [84]. The beating mechanism of the axoneme is produced by applying forces to each dynein motor, resulting in sliding movements based on their curvature and positional changes. The 3D cell path is the composite path of all dynein motors in 3D space. By utilizing the finite element method and solving the Frenet equations, we converted applied forces into positional and rotational changes for each dynein motor, thus generating the beating pattern observed in the sperm geometrical model [55]. The mesh densities of the different models vary between cells. This variability can affect the simulation runtime but does not significantly change the outcome measures (less than 3%). Additionally, the simulations achieved high resolution, shorter runtimes, and memory efficiency with an active dynein motor count of (N = 100) elements. Increasing the number of nodes did not affect the beating pattern of the sperm model.

### 2.7. Computerized Fluid Dynamic Simulation

We assumed a viscous fluid inside the different 3D fallopian tubes to describe the biophysical environment-mimicking system. The fluid has the same thermophysical properties as the cervical mucus, which is located inside the human fallopian tube (see Table 1) [80,85].

Navier–Stokes equations were solved to describe the cervical mucus flow, using computerized fluid dynamic (CFD) analysis software by SolidWorks flow simulation (Premium 2022 x64 Edition, Waltham, MA, USA). These equations are described by formulations of mass, momentum, and energy conservation laws [86].

The sperm and oocyte models were assumed to be neo-Hookean constitutive models and uncompressible materials. The sperm model was considered to be a hyperelastic material and hyperactivated model [56]. Hyperactivation is a sperm behavior associated with excessive sperm movement patterns observed in sperm during fertilization in mammals, characterized by a large and asymmetrical beating pattern of the flagellum. Here, the 3D hyperactivated beating patterns of different models were generated by increasing the dynein sliding forces applied to the sperm models $\left(F_{non-hyperactivated}=2{\times 10}^{-6}\;\mu N,F_{hyperactivated}=5\times 10^{-6}\;\mu N\right)$.

We tracked the sperm and oocyte models’ dynamics inside the viscous fluid in the normal and endometriosis fallopian tube models, and the numbers of models and simulations are given in Table 2. We assigned random initial positions for 10,000 sperm models within the intramural portion of each fallopian tube model. The sperm models were free to swim inside the fallopian tube from the intramural part to the fertilization site, the ampulla. We also analyzed the 3D paths of 100 oocyte models inside each fallopian tube by performing 100 individual simulations. For each simulation, a single oocyte model was initially positioned at the infundibulum portion and allowed to move freely toward the intramural portion within each fallopian tube model. The simulation was repeated 100 times, with the oocyte model placed in a different initial position for each iteration. To map the 3D trajectory of each oocyte, we observed its positions within various 3D fallopian tube models for 80 h. Specifically, we tracked the coordinates (*x*, *y*, *z*) of the central oocyte model as it moved within these models, and then integrated this positional data to create the comprehensive 3D trajectory for each oocyte model. The dynamic simulations were performed using SolidWorks 2023, Blender 2.91, and Python 3.3 SW (3D modeling and rendering package). Table S1 provides a comprehensive overview of the sequential steps involved in modeling the movement of sperm and oocytes within the different fallopian tube models. It delineates the intricate process involved in creating these 3D representations, specifying the simulation assumptions and detailing the software utilized at each stage of the modeling process.

## 3. Results

**Figure 4:**
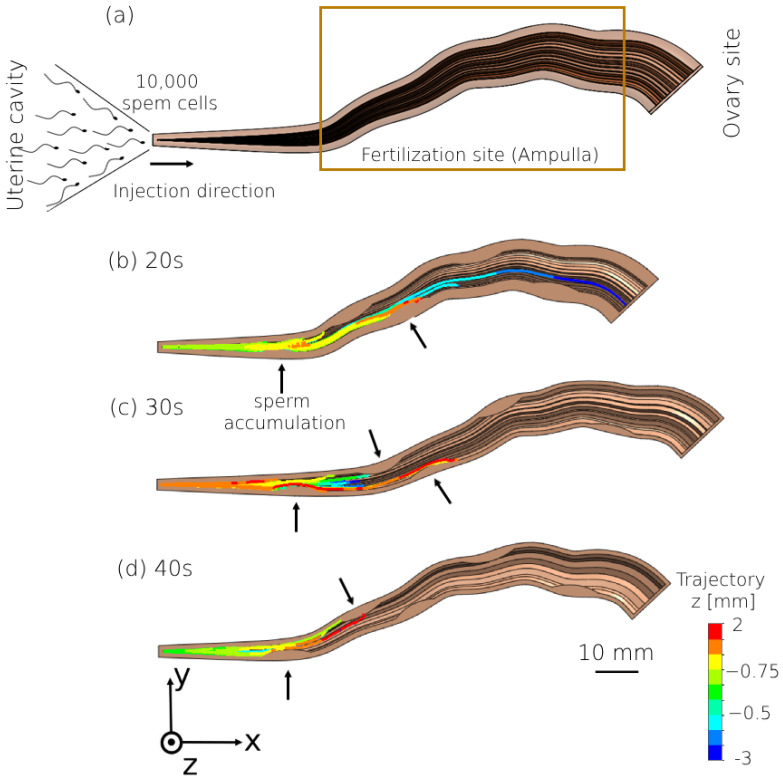
Swimming patterns of the normal sperm models in the different tubal endometriosis models. (**a**) Computerized fluid dynamic simulation design of the sperm cell model flow. Two-dimensional trajectories in the anterior view of the fallopian tube models for women in their (**b**) 20s, (**c**) 30s, and (**d**) 40s. The arrows indicate the presence of accumulated sperm models around the endometriosis lesions.

We analyzed the effect of tubal endometriosis on sperm cell transport by tracking their trajectories while swimming inside the tubal endometriosis models for the different age groups. Figure 4 displays the 2D trajectories of the swimming patterns of the sperm models inside each tubal endometriosis model. The trajectory contours delineate the positions of the sperm models within the sagittal plane (*z*-plane). These models were initially positioned at the origin point (0,0,0) and had the freedom to swim within the positive direction in the frontal plane (*x*, *y*) and in the sagittal plane (*z*), with coordinates capable of being positive or negative. We observed that most of the sperm models accumulated in the intramural and isthmus portions, where the first endometriosis lesion is located. This region is considered a critical barrier due to the narrow swimming area, known as the luminal area. The number of sperm models that succeeded in passing this narrow region and entering the ampulla decreased with the women’s aging. Moreover, the sperm models swam for shorter distances inside the ampulla as age increased. As shown in Figure 4b–d, sperm accumulations are seen around the endometriosis lesions inside the ampulla, especially around lesions 2 and 3. Hence, besides the women’s aging, tubal endometriosis affects the sperm cells’ transport inside the fallopian tube toward the oocyte. Video S2 shows sperm cell models swimming in the peristalsis–ciliary flow of the normal fallopian model for women in their 20s.

**Figure 5:**
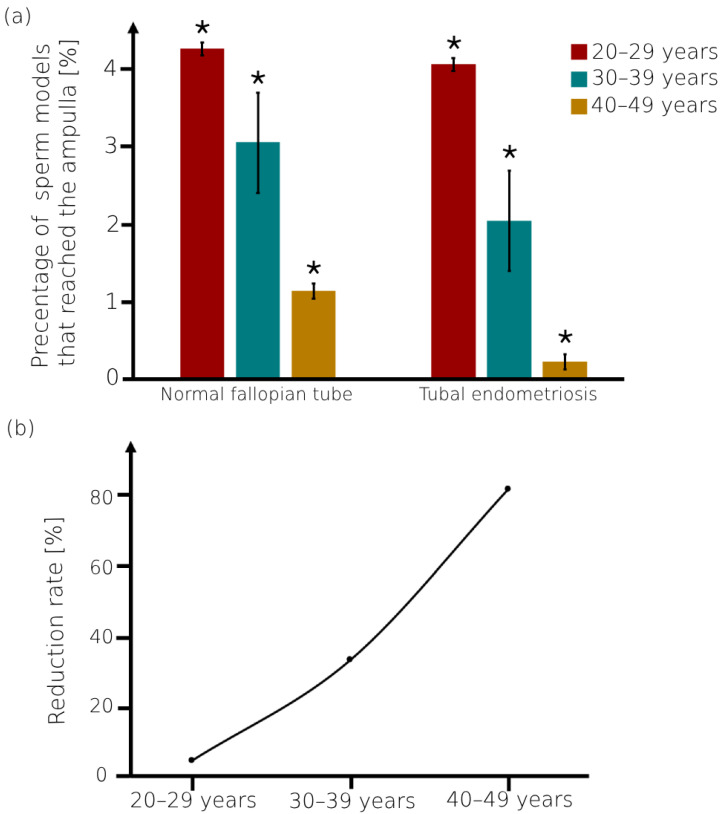
The normal sperm models at the fertilization site, the ampulla. (**a**) Percentage of the sperm models inside the ampulla of the normal fallopian tube and tubal endometriosis models for the different women’s age groups. Bars are mean ± standard error of the mean (SEM). (*) Significantly different from the normal control group (*p* < 0.05). (**b**) The rate of reduction of the number of sperm cells reaching the ampulla due to tubal endometriosis.

We described the effect of tubal endometriosis on the quantity of sperm models in the ampulla. Figure 5 presents a comparison between the percentages of the normal sperm models in the ampulla of tubal endometriosis models and the results from our earlier study on normal fallopian tube models [56]. For the normal fallopian tube and tubal endometriosis models, the percentage of the normal sperm models that succeeded in reaching the ampulla is the largest in women in their 20s, and it decreased with the women’s aging. For all ages, the number of sperm models inside the ampulla of the normal fallopian tube is the largest compared to their number in the tubal endometriosis (see Figure 5a). These findings correspond with prior research highlighting reduced progressive motile sperm cells due to tubal endometriosis [41,48,49] and women’s aging [26,30,31,37]. We also calculated the reduction rate of the number of sperm models in the ampulla due to tubal endometriosis for all different age groups (see Figure 5b). The reduction rate increased with age, reaching approximately 4.8% for women in their 20s and rising to 33.3% and 81.8% for women in their 30s and 40s, respectively. With women’s aging, fewer sperm cells succeed in reaching the fertilization site in cases of tubal endometriosis, especially for women in their 40s.

**Figure 6:**
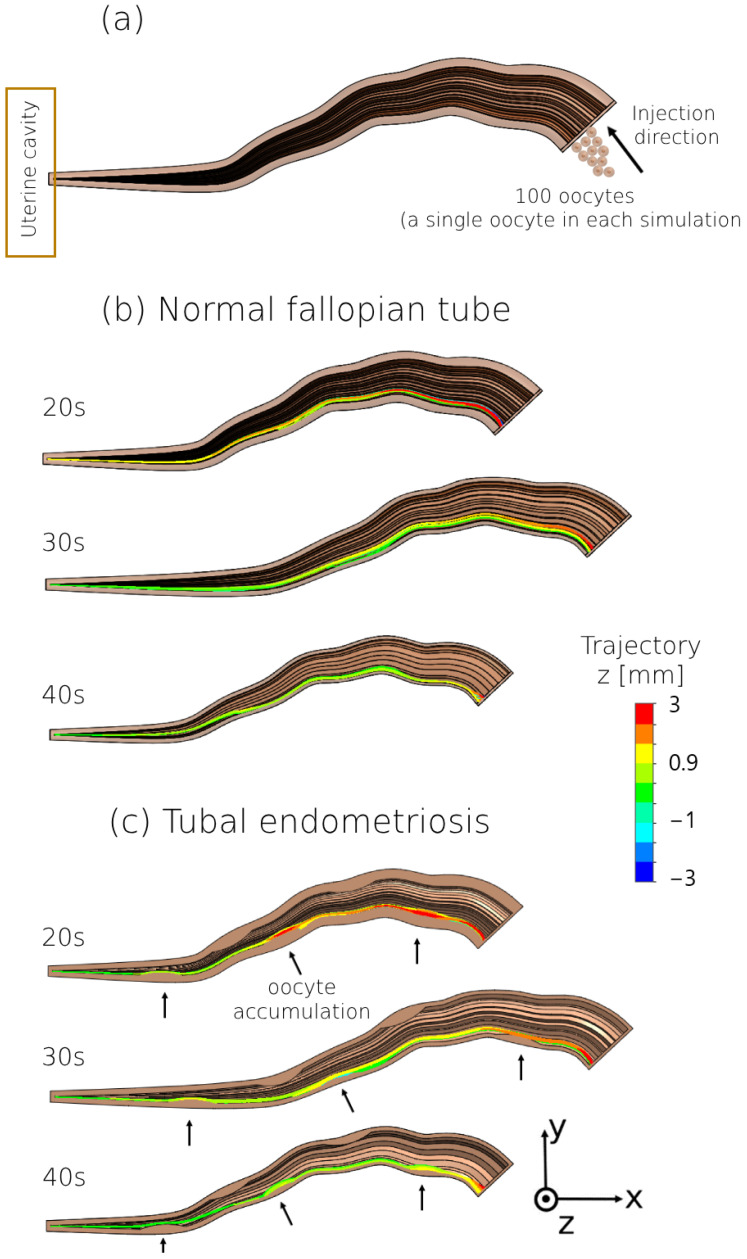
Swimming patterns of the oocyte fallopian tube models. (**a**) Computerized fluid dynamic simulation design of the oocyte model flow. Two-dimensional trajectories in the anterior view of the (**b**) normal fallopian tube and (**c**) tubal endometriosis models for women in their 20s, 30s, and 40s. The arrows indicate the presence of accumulated oocyte models around the endometriosis lesions.

We also tracked the oocyte models during swimming inside the normal fallopian tube and tubal endometriosis models, for all age groups (Figure 6). The oocyte models exhibited forward movement toward the uterine cavity with minimal lateral displacement in both fallopian tube models, regardless of endometriosis presence. Most oocyte models successfully reached the uterine cavity in both fallopian tube models, although fewer became stuck in the mucosal layer of the normal fallopian tube compared to those affected by tubal endometriosis. Accumulations of oocyte models were observed around the endometriosis lesions in the ampulla, especially lesions 3 and 5 (Figure 6a–c). However, as women aged, there was an increase in the percentage of oocyte models that became stuck and failed to reach the uterine cavity. Therefore, tubal endometriosis may interfere with the ability of the oocyte to reach the uterine cavity, especially with women’s aging. Video S3 provides a visual representation of oocyte swimming within the peristalsis–ciliary flow of the normal fallopian model for women in their 20s.

**Figure 7:**
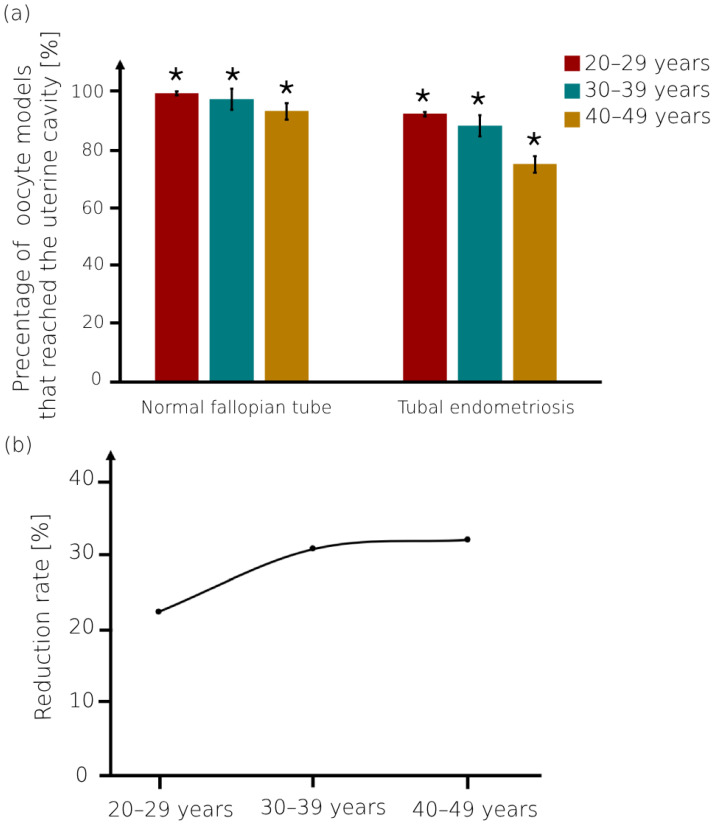
The oocyte models that reached the uterine cavity. (**a**) Percentage of the oocyte models that succeeded in passing through the normal fallopian tube and tubal endometriosis models and reaching the uterine cavity for the different women’s age groups. Bars are mean ± standard error of the mean (SEM). (*) Significantly different from the normal control group (*p* < 0.05). (**b**) The reduction rate of the oocytes that reached the uterine cavity due to tubal endometriosis.

We calculated the number of oocyte models that reach the uterine cavity while moving in both the normal fallopian tubes and tubal endometriosis models (Figure 7). For all ages, the percentages of the oocyte models that succeeded in passing through the normal fallopian tube and reaching the uterine cavity were the largest (92–98%) compared to those in the tubal endometriosis models (74–91%). Consequently, tubal endometriosis led to an increased number of oocyte models that became stuck inside the fallopian tube (Figure 7a). Additionally, for women in their 20s, the percentages of the oocyte models that reached the uterine cavity were the largest in both the normal fallopian tube (98% ± 0.7%) and tubal endometriosis (91% ± 0.7%) compared to other age groups. However, with increasing age, a decrease in the number of oocyte models was observed in both normal and pathological fallopian tubes (Figure 7a). Figure 7b demonstrates the reduction rate of the oocyte models that reached the uterine cavity due to tubal endometriosis. For women in their 20s with tubal endometriosis, there was a reduction of approximately 22% in the number of oocytes that successfully passed the fallopian tube. Moreover, as age increased, the number of oocytes decreased due to tubal endometriosis by approximately 30% and 32% for women in their 30s and 40s, respectively. Previous studies have highlighted the association of tubal endometriosis with an increased risk of impaired oocyte transportation within the fallopian tube, especially with women’s aging [50,51,52,53].

## 4. Discussion

We presented a 3D dynamic mechanical model describing the dynamics of sperm cells and oocytes within the fallopian tubes, addressing the influence of both tubal endometriosis and women’s aging on their behavior. Our method simulates the intricate processes inside the fallopian tube to better understand fertility-related sperm cell and oocyte transportation mechanisms. We implemented 3D geometrical models of sperm cell and female oocyte dynamics through the normal fallopian tube and tubal endometriosis models, for different women’s age ranges. Ciliary activity and bidirectional uterine peristalsis were included inside the normal fallopian tube and tubal endometriosis models, simulating the biomechanics of the inner tube walls. This integration provides insights into the behavior of sperm cells and oocytes in normal and endometriosis-affected fallopian tubes across different women’s age ranges. By various dynamic simulations, we quantified the percentage of sperm models that reached the fertilization site within the fallopian tube models. Additionally, we measured the percentage of oocyte models that successfully reached the uterine cavity site. This observation period spanned 80 h and was conducted in both normal and endometriosis-affected fallopian tube models.

In contrast to previous works (e.g., [56]), the present study has incorporated endometriosis and examined the combined impact of a women’s age and endometriosis on the behavior of sperm cells within the fallopian tubes. Additionally, for the first time, we have added the movement of oocytes within fallopian tubes and checked how they are affected by endometriosis across different age groups. Furthermore, we examined the relationship between the increased chances of ectopic pregnancy and women’s aging, especially in tubal endometriosis cases.

Some limitations associated with our 3D models and simulation definitions may influence the obtained results. First, in spite of our efforts to match previous 3D acquisitions, the geometry of our 3D models might not fully capture the complexities of the real biological and physiological system. Additionally, the initial positions of the sperm and oocyte models within the fallopian tube were randomly assigned to physiologically reasonable starting points, introducing variability that could affect trajectories and interactions during simulation. To address this, we repeated each simulation multiple times with different physiologically reasonable initial positions. The variability in results is presented in Figure 5 and Figure 7 as the standard deviation (SD) of the average number of sperm and oocyte models reaching their respective goal sites. The movement patterns of sperm cells and oocytes within fallopian tubes affected by endometriosis for different age groups were within the theoretical range reported in the literature. Our study observed that due to endometriosis and increasing age, there is a 40–80% decrease in the number of motile sperm cells, aligning with previous clinical reports [41,48,49]. Additionally, this condition directly increases the chances of ectopic pregnancy up to 2.6 times, which is consistent with earlier clinical meta-analyses [51,54]. Comparison between these different simulation results sheds light on the influence of tubal endometriosis and women’s aging on fertilization and successful pregnancy rates. The beneficial educational impacts of 3D simulation and biomarkers can enhance the authenticity of learning and deepen the learner’s anatomical understanding of disease processes. Clinical outcomes could be improved through increased awareness of the applied and translational aspects of basic research, as discussed in a previous study [87]. Clinical studies face significant challenges when attempting to explore the dynamics within the fallopian tube, primarily due to complexities in monitoring sperm cell and oocyte movement in real time. The internal nature and small size of the fallopian tubes make direct observation difficult, compounded by dynamic changes in fluid dynamics and hormonal influences. Traditional imaging methods offer limited visibility, and ethical considerations restrict invasive procedures. However, advancements in imaging and computational modeling offer promise for overcoming these obstacles and enhancing our understanding of fallopian tube dynamics. For this reason, we aligned our findings with meta-analysis studies that have delved into the effects of endometriosis on sperm motility and the increased risk of ectopic pregnancy, especially as women age. This research represents a valuable tool in fertility assessment and contributes to a deeper comprehension of the impact of age-related morphological and mechanical changes and tubal endometriosis on fertility.

Tubal endometriosis affects the transport of sperm cells within the fallopian tube across different age groups. For all age groups, a reduced number of sperm cells arrived at the fertilization site within the fallopian tube in endometriosis compared to the normal fallopian tube. Furthermore, most sperm cells tend to accumulate around the endometriosis lesions in the fallopian tube, notably in the isthmus portion and the beginning of the ampulla region. This accumulation occurred due to the decrease in the luminal area as a result of the endometriosis lesions. The narrowing of the luminal area restricts the available swimming space of the sperm cells and leads to their accumulation caused by more cell-to-cell and cell-to-surface collisions inside the fallopian tube [88,89,90,91,92]. Cell-to-cell and cell-to-surface collisions in the fallopian tube hinder sperm mobility and lead to accumulation. These collisions impede sperm movement, causing them to slow down or change direction, making it challenging to reach the fertilization site. Additionally, repeated collisions can cause sperm to cluster together or adhere to the tube wall, further impeding their progress. As a result, sperm accumulation occurs, reducing their mobility within the fallopian tube. These results align with prior studies indicating a decline in progressive motile sperm cells due to tubal endometriosis [41,48,49,93,94].

The impact of tubal endometriosis on sperm transport within the fallopian tube is substantial and amplifies with the women’s age. With aging, we observed a decrease in the number of sperm cells reaching the fertilization site in both normal fallopian tubes and those affected by tubal endometriosis, particularly noticeable in women in their 40s. As women age, the inner muscle layer of the fallopian tubes stiffens, increasing friction between the cells and the fallopian tube walls, which hinders the forward movement of the sperm cells, increasing resistance and slowing down their velocity. This impedes their ability to navigate through the fluid medium, leading to decreased efficiency in reaching the desired destination within the female reproductive tract. Consequently, friction adversely affects sperm motility and overall fertility potential. This indicates that as women age, the efficiency of sperm transport within the fallopian tube is compromised, especially in cases of tubal endometriosis. These findings are equivalent to earlier reports indicating compromised sperm transportation within the fallopian tube due to women’s aging, subsequently impacting fertilization potential [26,30,31,37].

We also presented the reduction rates of sperm cells within the fertilization site resulting from the combined effects of women’s aging and tubal endometriosis. Hence, older women affected by tubal endometriosis could display a reduced presence of sperm cells within the fertilization site of their fallopian tube, possibly impacting fertilization rates. With a decreased presence of sperm cells at the fertilization site, the chance of sperm encountering and fertilizing an oocyte diminishes, thereby reducing the chances of successful fertilization. Additionally, reduced competition among sperm cells may lead to suboptimal fertilization, as fewer opportunities for the selection of the most viable sperm cell arise. Moreover, fewer opportunities for effective sperm–oocyte binding to the zona pellucida surrounding the egg may contribute to decreased fertilization rates. Lastly, suboptimal fertilization rates resulting from fewer sperm cells at the fertilization site can have cascading effects on subsequent stages of reproduction, including embryo development, implantation, and the establishment of a successful pregnancy. Our results hold considerable significance in comprehending the dynamics of sperm behavior concerning women’s aging and fallopian tube defects related to endometriosis.

Tubal endometriosis significantly impacts the transport mechanism of the female oocyte inside the fallopian tube for the different women’s age groups. The observations indicated a forward movement of oocyte models towards the uterine cavity with small lateral displacements in the different fallopian tube models, irrespective of the presence of endometriosis. For all ages, most of the oocyte models successfully reached the uterine cavity; nevertheless, some became stuck in the inner wall of the fallopian tube, especially in tubal endometriosis cases. Therefore, a fallopian tube affected by endometriosis is associated with a decrease in the number of female oocytes that successfully reach the uterine cavity. We also observed a further decline in the number of oocyte models that successfully passed the entire fallopian tube and reached the uterine cavity due to the women’s aging. The efficient transport of fertilized oocytes from the fallopian tubes to the uterine cavity is crucial for timely embryo implantation. Any disruption or decrease in this transport process can delay implantation or result in implantation failure, further impacting fertility outcomes. Additionally, the uterine cavity provides an optimal environment for embryo implantation and development. With fewer fertilized oocytes reaching this site, the chances of successful implantation decrease, thereby reducing the likelihood of pregnancy establishment. Overall, the diminished presence of fertilized oocytes in the uterine cavity significantly affects fertility by lowering the chances of successful embryo implantation, subsequent development, and pregnancy success.

Hence, women’s aging not only affects sperm cell motility, it also significantly impacts the dynamics of oocyte transport within the fallopian tube, especially in tubal endometriosis cases. Impaired oocyte transport within the fallopian tube affected by endometriosis could hinder the meeting between sperm and oocyte and lead to potential fertilization failure. Furthermore, if fertilization occurs within a fallopian tube affected by endometriosis, there is a risk that the fertilized oocyte might become stuck and implant itself within the inner walls of the fallopian tube. This condition is known as an ectopic pregnancy, where the fertilized oocyte cannot survive or develop into a baby. This situation can result in life-threatening bleeding due to the growing tissue. Our findings have shown an increased risk of oocyte entrapment within the fallopian tube affected by endometriosis, which can increase the risk of ectopic pregnancy. These results align with previous studies concluding that tubal endometriosis is associated with infertility issues and an increased risk of ectopic pregnancy [51,54].

Finally, endometriosis and aging significantly affect the transport of sperm cells and oocytes within the fallopian tubes. Endometriosis can cause blockages that restrict movement, leading to collisions and accumulation of cells, reducing the number reaching their intended destinations. Aging stiffens the inner muscle layer of the tubes, increasing friction and reducing the inner diameter, which also impedes cell movement and causes accumulation. This results in fewer sperm cells and oocytes reaching the fertilization site or the uterine cavity, decreasing the likelihood of fertilization and impacting fertility. Our research focused on a clinically average population under uniform conditions. Future studies should explore variations in sperm integrity, quantity, and female reproductive system morphology for a more comprehensive understanding. We aimed to fill a research gap by examining the combined effects of endometriosis and aging on female fertility and oocyte dynamics, an area less studied compared to sperm motility. Our findings indicate that endometriosis and aging alter the tubal environment, reducing the chances of successful fertilization. Endometriosis lesions can obstruct the passage of fertilized oocytes, leading to timing mismatches with the uterine environment and increasing the risk of ectopic pregnancy. Prolonged retention of oocytes near endometriosis lesions can create adverse conditions, leading to oocyte degeneration. These insights help diagnose fertility problems more accurately and develop personalized treatments for women of different ages with or without tubal endometriosis, improving pregnancy outcomes and infertility management. Our study uses a comprehensive model to simulate sperm and oocyte movement within fallopian tubes under various conditions, employing the finite element method to examine the impact of endometriosis and aging. While fertility studies have traditionally relied on clinical, microfluidic, and theoretical research, our utilization of advanced computer modeling methods provides a complementary perspective [45,46,47,48,49,50,51,52,53,54]. We believe our findings enrich the research in this field, thus contributing to a more holistic understanding of fertility dynamics.

## 5. Conclusions

To conclude, our study introduces an advanced dynamic mechanical model that assesses the influence of tubal endometriosis and women’s aging on the motility of healthy human sperm cells and oocytes within the female fallopian tubes. This model describes the morphological and mechanical changes in the fallopian tube attributed to tubal endometriosis and women’s aging and clarifies their impact on fertilization potential. Our methodology integrates comprehensive dynamic 3D modeling of human sperm cells and oocytes moving in different 3D fallopian tube configurations and their interactions with real internal environmental factors. This approach opens doors for further investigation of the effects of tubal endometriosis and age on fertilization and pregnancy success, potentially bridging gaps in earlier research. Specifically, our observations of sperm cell and oocyte motility within the fallopian tube for different women’s age groups present a novel direction for providing new diagnostic and treatment tools for tubal endometriosis disease and age-related infertility issues. Moreover, our 3D dynamic model could have profound implications for the diagnosis and treatment of fertility issues associated with tubal endometriosis cases and age-related changes in women and for personalized medicine predictive simulations before treatment. Future studies will aim to provide deeper insights into the intricate biological interactions and mechanical processes that occur during fertilization. Understanding the fertilization process by developing a comprehensive biomechanical model could have significant implications for reproductive biology and medical interventions in fertility treatments. In the future, the suggested technique might find use for personalized medicine for in silico testing of possible treatments for endometriosis and age-related problems.

## Figures and Tables

**Table 1:** Thermophysical properties of the cervical mucus.

Density	$1007\;\left[\frac{kg}{{mm}^{2}}\right]$
Specific heat	$4140\;\left[\frac{J}{kJ*K}\right]$
Thermal conductivity	$0.627\left[\frac{W}{mK}\right]$
Dynamic viscosity	$\left(0.02\gamma +0.98\right)[Pa*s]$

**Table 2:** Computerized Fluid Dynamic Simulation Design.

Numbers of Simulations for Women’s Age Group	Total Number of Sperm/Oocyte Models in a Single Simulation	With/Without Endometriosis
1	10,000 sperm models	Without
1	10,000 sperm models	With
100	1 single oocyte model	Without
100	1 single oocyte model	With

## Data Availability

All data are available from the corresponding author upon a reasonable request.

## Supplementary Materials

The following supporting information can be downloaded at: https://www.mdpi.com/article/10.3390/bioengineering11080852/s1, Figure S1: The 3D geometrical model of the fallopian tube with (a) three, (b) seven, and (c) ten endometriosis lesions for women in their 20s. Table S1: Computerized 3D modeling and fluid dynamic simulation design. Video S1: A single tubal contraction of the fallopian tube model for women in their 20s. Video S2: Sperm cells swim within the peristalsis–ciliary flow of the normal fallopian model for women in their 20s. Video S3: An oocyte movement within the peristalsis–ciliary flow of the normal fallopian tube model for women in their 20s.
