# Supplementary material for: The Synergic Effect of Tubal Endometriosis and Women’s Aging on Fallopian Tube Function: Insights from a 3D Mechanical Model
Source: Bioengineering (Basel). 2024 Aug 20;11(8):852. doi: 10.3390/bioengineering11080852 (PMC11351632; doi:10.3390/bioengineering11080852)
Supplement: Supplementary file 1 [file bioengineering-11-00852-s001.zip › bioengineering-3108538-supplementary.pdf]

Supplementary Materials

The Synergic Effect of Tubal Endometriosis and Women's Aging on Fallopian Tube Function: Insights from a 3D Mechanical Model

Mayssam Nassir, Mattan Levi and Natan T. Shaked *

Department of Biomedical Engineering, Faculty of Engineering, Tel Aviv University, Tel Aviv 69978, Israel; mayssam.nassir@gmail.com (M.N.); mattanlevi@gmail.com (M.L.)
* Correspondence: nshaked@tau.ac.il

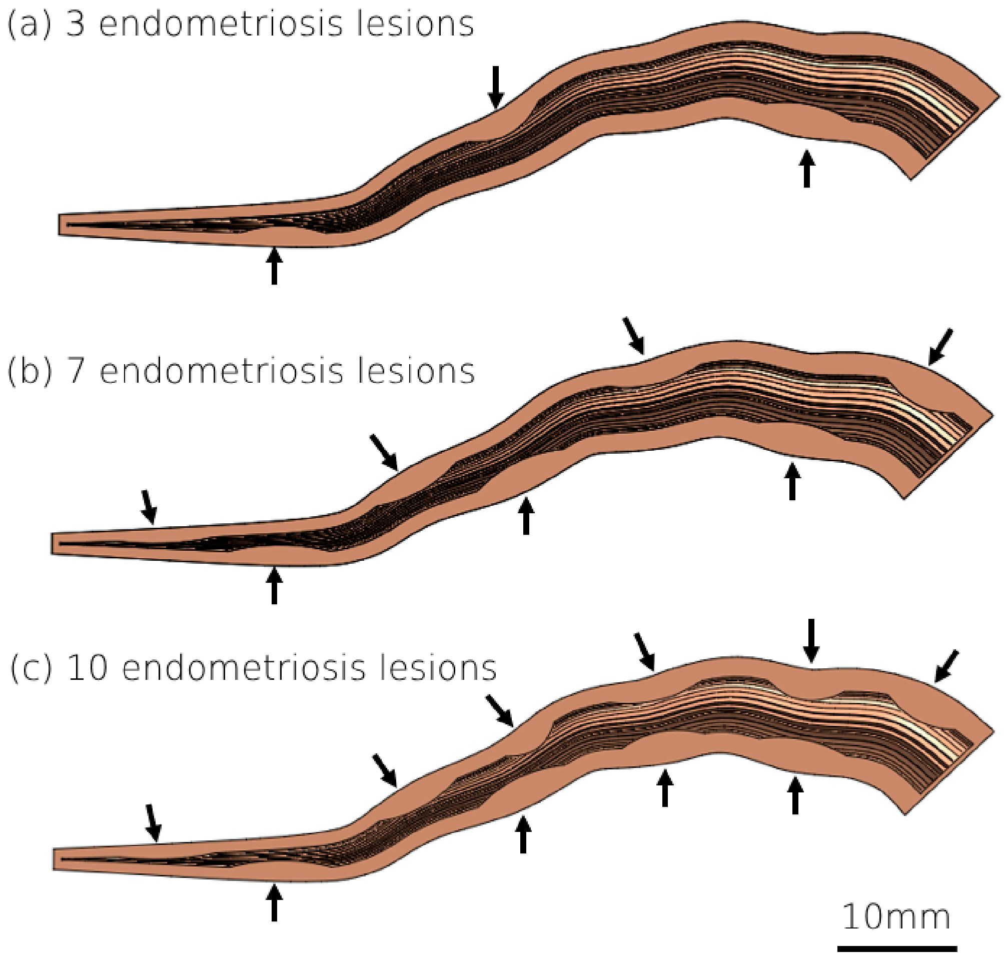

Figure S1. 3D geometrical model of the fallopian tube with (a) three, (b) seven, and (c) ten endometriosis lesions for women at their 20s.

Table S1. Computerized 3D modeling and fluid-dynamic simulations design.

<table>
<tr><th rowspan="2">Model</th><th rowspan="2">3D model
(drawing and reconstructing in SOLIDWORKS)</th><th colspan="3">Flow simulations
(using Computerized Fluid Dynamic (CFD) analysis software by SolidWorks flow simulation)</th><th colspan="2">Data analysis
(using Python 3.3 SW and Blender 2.91)</th></tr>
<tr><th>Internal movement</th><th>Model assumptions</th><th>Flow and dynamics assumptions</th><th>3D path data</th><th>Drawing 3D trajectories and movement recording</th></tr>
<tr><td>Fallopian tube</td><td rowspan="4">We employed real images and geometrical parameters obtained from actual measurements performed by MRI, US, or optical experiments of the human fallopian tube, oocyte, ciliated cell and sperm cell. Based on this data, we created 2D projections in the sagittal, coronal, and transverse planes, and then reconstructed the 3D geometry for each model.</td><td>Implementing a vertical force as a function of distance and time on the internal layer.</td><td>The mucosal layer consists of isotropic compressible materials and is modeled using a Neo-Hookean constitutive model with a strain energy function.</td><td rowspan="5">1. The fallopian tubes are filled with fluid.
2. Tubal contraction is applied to the internal layer of the fallopian tube models.
3. Sperm and oocyte models are allowed to freely swim within the fluid.
4. The fluid is incompressible, with a constant density throughout the flow field.
5. The fluid adheres to Newton's law of viscosity.
6. Flow is maintained in a steady state, where flow properties remain constant over time.
7. Interactions between sperm/oocyte and the tube walls occur within a hydrodynamic environment generated by peristaltic contractions and ciliary motion. The models do not adhere to the internal wall and do not exhibit chemotactic responses.</td><td rowspan="5">The 3D coordinates of the sperm and oocyte models inside the fallopian tube models were acquired through Computational Fluid Dynamics (CFD) analysis software by SolidWorks flow simulation.</td><td rowspan="5">1. The 3D paths of each sperm and oocyte model, along with the quantification of their occurrences at various sites within each fallopian tube model, were achieved using an algorithm developed in Python 3.3 SW.
2. The dynamics of sperm and oocyte models within different fallopian tube models were recorded using Blender 2.91.</td></tr>
<tr><td>Sperm cells</td><td>Applying forces to the flagellum, inducing sliding movements contingent on their curvature and positional changes.</td><td>1. Neo-Hookean constitutive models and incompressible materials.
2. Hyper-elastic and hyperactivated model.</td></tr>
<tr><td>Oocyte</td><td>Motion of a spherical object within a fluid under the influence of fluid parameters.</td><td>Neo-Hookean constitutive models and incompressible materials.</td></tr>
<tr><td>Ciliated cells</td><td>Enforcing forces to execute ciliary beating.</td><td></td></tr>
<tr><td>Fluid</td><td></td><td>Utilizing Navier-Stokes equations to model the flow of cervical mucus.</td><td>Identical thermo-physical properties to cervical mucus of human fallopian tube.</td></tr>
</table>
